# Dental Poly(methyl methacrylate)-Based Resin Containing a Nanoporous Silica Filler

**DOI:** 10.3390/jfb13010032

**Published:** 2022-03-15

**Authors:** Kentaro Hata, Hiroshi Ikeda, Yuki Nagamatsu, Chihiro Masaki, Ryuji Hosokawa, Hiroshi Shimizu

**Affiliations:** 1 Division of Oral Reconstruction and Rehabilitation, Department of Oral Functions, Kyushu Dental University, Kitakyushu 803-8580, Japan; r17hata@fa.kyu-dent.ac.jp (K.H.); masaki@kyu-dent.ac.jp (C.M.); hosokawa@kyu-dent.ac.jp (R.H.); 2 Division of Biomaterials, Department of Oral Functions, Kyushu Dental University, Kitakyushu 803-8580, Japan; yuki-naga@kyu-dent.ac.jp (Y.N.); r14shimizu@fa.kyu-dent.ac.jp (H.S.)

**Keywords:** methacrylates, acrylic resins, silicate, composite resins, dental materials

## Abstract

Poly(methyl methacrylate) (PMMA)-based resins have been conventionally used in dental prostheses owing to their good biocompatibility. However, PMMA-based resins have relatively poor mechanical properties. In the present study, a novel nanoporous silica filler was developed and introduced into PMMA-based resins to improve their mechanical properties. The filler was prepared by sintering a green body composed of silica and an organic binder, followed by grinding to a fine powder and subsequent silanization. The filler was added to photocurable PMMA-based resin, which was prepared from MMA, PMMA, ethylene glycol dimethacrylate, and a photo-initiator. The filler was characterized by scanning electron microscopy (SEM), X-ray diffraction analysis, nitrogen sorption porosimetry, and Fourier transform infrared (FT-IR) spectroscopy. The PMMA-based resins were characterized by SEM and FT-IR, and the mechanical properties (Vickers hardness, flexural modulus, and flexural strength) and physicochemical properties (water sorption and solubility) were evaluated. The results suggested that the filler consisted of microparticles with nanopores. The filler at 23 wt % was well dispersed in the PMMA-based resin matrix. The mechanical and physicochemical properties of the PMMA-based resin improved significantly with the addition of the developed filler. Therefore, such filler-loaded PMMA-based resins are potential candidates for improving the strength and durability of polymer-based crown and denture base.

## 1. Introduction

Polymethylmethacrylate (PMMA)-based resins are widely used in dental applications because of their excellent biocompatibility, stable physicochemical properties, easy manipulation, low cost, and appropriate aesthetics [1,2,3]. PMMA-based resins are commonly used for dental applications, such as denture bases, denture teeth, temporary crowns, provisional restorations, adhesives, and orthodontic retainers. However, clinical problems related to the use of PMMA-based resins include fracture and wear owing to their insufficient mechanical properties [4,5]. Improving the mechanical properties of PMMA-based resins is a critical issue for their long-term use in the oral environment without early failures.

To improve the mechanical properties, many studies have introduced fillers or fibers into a PMMA-based resin matrix [6,7]. A systematic review paper suggested that the addition of filler to the resin matrix can increase mechanical properties such as flexural strength, flexural (elastic) modulus, and fracture toughness [7]. For instance, the addition of microcrystalline cellulose at 5 wt % reinforced the PMMA-based resin in terms of increasing the flexural strength and modulus [8]. A double-modified organoclay was incorporated in the PMMA matrix at 0.5 wt %, resulting in increased flexural strength, flexural modulus, and fracture toughness [9]. Silanized alumina micro-particles with 0.1 wt % increased the flexural strength of PMMA-based resin [10]. ZrO_2_ nanotubes were added to a PMMA-based resin, which increased its flexural strength [11]. Furthermore, the addition of silanized silica nanoparticles increased the flexural strength, flexural modulus, and fracture toughness of the PMMA-based resin [12]. Thus, the addition of fillers at an appropriate amount can reinforce the mechanical properties of PMMA-based resins.

However, the addition of large amounts of fillers often has adverse effects on the mechanical properties of PMMA-based resins. Excess filler (usually above approximately 5 wt %) degrades the mechanical properties of the PMMA-based resin, because the filler forms aggregates and voids in the resin matrix [7]. For instance, the flexural strength of heat-polymerized PMMA-based resin increased with increasing nano-filler (silica, alumina, and zirconia) content up to 1 wt %, and decreased above 5 wt % [13]. The flexural strength of the PMMA-based resin containing 5 wt % silica nanoparticles was lower than that of the pure resin [14]. The flexural strength of a PMMA-based resin increased with the addition of fluoridated glass filler up to 5 wt %, and then decreased [15]. 

To overcome this issue, the present study focused on the development of porous fillers to improve the mechanical properties of PMMA-based resins. Porous filler containing continuous internal pores can form a mechanically interlocked structure at the interface between the infiltrated resin and filler surface [16]. Porous fillers have the potential to avoid degradation of the properties of the PMMA-based resin when a large amount of filler is added. In resin composites, some types of porous fillers have been introduced to improve the mechanical and physicochemical properties of resins [17,18,19,20,21]. For instance, a nanoporous silica filler prepared by thermal sintering increased the flexural strength, flexural modulus, and fracture toughness of copoly(bisphenol A-glycidyl methacrylate (Bis-GMA)/Triethylene glycol dimethacrylate (TEGDMA)) by adding 70 wt % of filler [19]. A wrinkled mesoporous silica improved the mechanical properties, including the flexural strength, flexural modulus, compressive strength, and Vickers microhardness of copoly(Bis-GMA/TEGDMA) [20]. A dendritic porous silica filler added at 36 wt % showed the best reinforcing effect on the flexural strength, compressive strength, and work of fracture of copoly(Bis-GMA/TEGDMA) [21]. Similarly, it is expected that porous fillers have the potential to improve the mechanical properties of PMMA-based resins.

This study aimed to develop a novel nanoporous filler to enhance the mechanical properties of a PMMA-based resin. First, we demonstrate the preparation of a nanoporous filler possessing highly surface area. Then, the resultant filler was loaded with 23 wt % (the upper limit before deterioration in the properties was observed) into a photocurable PMMA-based resin, and its mechanical and physicochemical properties were examined to evaluate the feasibility of its use in dental restorative materials.

## 2. Materials and Methods

### 2.1. Materials

The reagents used for the preparation of all samples are listed in Table 1.

### 2.2. Preparation of Porous-Silica Filler

SiO_2_ nanoparticles (6 g) were dispersed in distilled water (54 g) at pH 3 by ultrasonication for 1 h. The resultant SiO_2_ suspension was mixed with 10 wt % poly(vinyl alcohol) (PVA) solution (11.4 g) using a magnetic stirrer at 30 °C for 24 h. The obtained slurry was poured into a container and dried in an oven at 30 °C for 7 days. The resultant green body was heat treated in an electric furnace at 600 °C for 3 h in air to burn out the PVA binder. The heat-treated body was pre-sintered at 950 °C for 1 h in air. The obtained monolith was crushed using a mortar and pestle. The resultant coarse powder was ground and simultaneously silanized in an ethanol medium containing 1 wt % of γ-MPTS using a ball-milling device with zirconia balls. The ground powder was dried in an oven at 80 °C for 24 h and then sieved through a 500 μm mesh. Finally, a silanized nanoporous silica filler was obtained.

### 2.3. Characterization of Nanoporous Silica Filler

The microstructure of the filler was observed using field-emission scanning electron microscopy (FE-SEM; S-4300, Hitachi High-Tech Corp., Tokyo, Japan). Prior to observation, the sample was coated with platinum via sputtering. The observation conditions were an acceleration voltage of 5 kV and a working distance of 15 mm.

The surface area and pore size distribution of the filler were estimated using the Brunauer–Emmett–Teller (BET) method and the Barrett–Joyner–Halenda (BJH) method, respectively, using nitrogen sorption–desorption curves measured by a porosimeter (QuadraSorb SI, Quantachrome Instruments, Boynton Beach, FL, USA).

The crystal phase of the filler was determined using X-ray diffraction (XRD; RINT 2100VLR/PC, Rigaku, Tokyo, Japan) with a Cu Kα X-ray source (λ = 1.5406 Å).

The silanization of the filler was examined by Fourier transform infrared (FT-IR) analysis using a spectrometer (IRSpirit, Shimadzu Corp., Kyoto, Japan) with a diffuse reflectance unit with a resolution of 4 cm^−1^.

### 2.4. Preparation of the Filler-Loaded PMMA-Based Resin

MMA, PMMA, and ethylene glycol dimethacrylate (EGDMA) were mixed at a weight ratio of 63:30:7 using a magnetic stirrer at 80 °C for 30 min. The resultant resin mixture was further mixed with the prepared filler (23 wt % relative to the resin) by ultrasonication for 30 min. Subsequently, the photo-initiator phenylbis (2,4,6-trimethylbenzoyl) phosphine oxide (BAPO, 0.5 wt %) was dissolved into the mixture using a stirrer. As a result, the liquid state of the filler-loaded PMMA-based resin was obtained. The resultant resin was poured into a transparent silicone mold with dimensions of 15 mm × 5 mm × 1.5 mm and light-cured using a light-irradiator (α-LIGHT II N, J. Morita Corp., Osaka, Japan) for 3 min. The cured resin was polished using emery paper up to #2000 and formed into a bar shape (14 mm × 4 mm × 1.2 mm) according to the ISO standard 10477: 2018 [22]. The bar-shaped samples were used for the following characterization experiments.

### 2.5. Characterization of the PMMA-Based Resins

The microstructure of the PMMA-based resin was observed using SEM under the same conditions as mentioned above. FT-IR analysis was conducted for the PMMA-based resins using a spectrometer with an attenuated total reflection (ATR) unit. The degree of conversion of the *C*=*C* bonds in the samples was estimated from the FT-IR spectra. In the FT-IR spectra, two characteristic bands at 1637 cm^−1^ (stretching of carbon double bond *C*=*C*) and 1720 cm^−1^ (stretching of carbonyl group *C*=*O*) before and after polymerization of the sample were used to calculate the degree of conversion using the following equation [23]:

(1) $$\mathrm{Degree}\;\mathrm{of}\;\mathrm{conversion}\;\left(\%\right)=\left(1-\frac{{\left[A\left(C=C\right)/A\left(C=O\right)\right]}_{polymer}}{{\left[A\left(C=C\right)/A\left(C=O\right)\right]}_{monomer}}\right)\times 100$$

where *A*(*C*=*C*) and *A*(*C*=*O*) are the band intensities. 

The mechanical properties of the PMMA-based resins were characterized by flexural strength, flexural modulus, and Vickers hardness tests according to the procedures described in a previous study [24]. The flexural strength and modulus were obtained by three-point bending tests using a universal testing machine (AGS-H, Shimadzu Corp., Kyoto, Japan) with a crosshead speed of 1 mm/s and a support span of 12 mm. After the three-point bending tests, the fractured samples were used for the Vickers hardness test. Each test was performed ten times for each group (n = 10).

The physicochemical properties (water sorption and water solubility) of the PMMA-based resins were measured using the procedures described in the ISO standard 10477: 2018 [22]. The sample was kept in an oven at 37 °C for seven days under dry conditions (initial weight, *m*1). The dried sample was immersed in distilled water at 37 °C for seven days (final weight, *m*2). The immersed sample was dried again in an oven for seven days (dried weight, *m*3). The following equations were used to calculate water sorption (ρ_ws_) and solubility (ρ_s1_):

(2) $$\rho_{\mathrm{ws}}=\frac{\left(m2-m3\right)}{V}$$

(3) $$\rho_{s1}=\frac{\left(m1-m3\right)}{V}$$

where *V* is the volume of the rectangular bar-shaped sample. Ten measurements were obtained for each group (n = 10).

### 2.6. Statistical Analysis

The results of the degree of conversion, mechanical properties, and physicochemical properties were compared between the PMMA-based resins with and without fillers by means of Student’s *t*-test using statistical software (EZR, Saitama Medical Center, Jichi Medical University, Saitama, Japan). The significance level was set to 0.01 for all results.

## 3. Results

### 3.1. Nano-Porous Silica Filler

**Figure 1:**
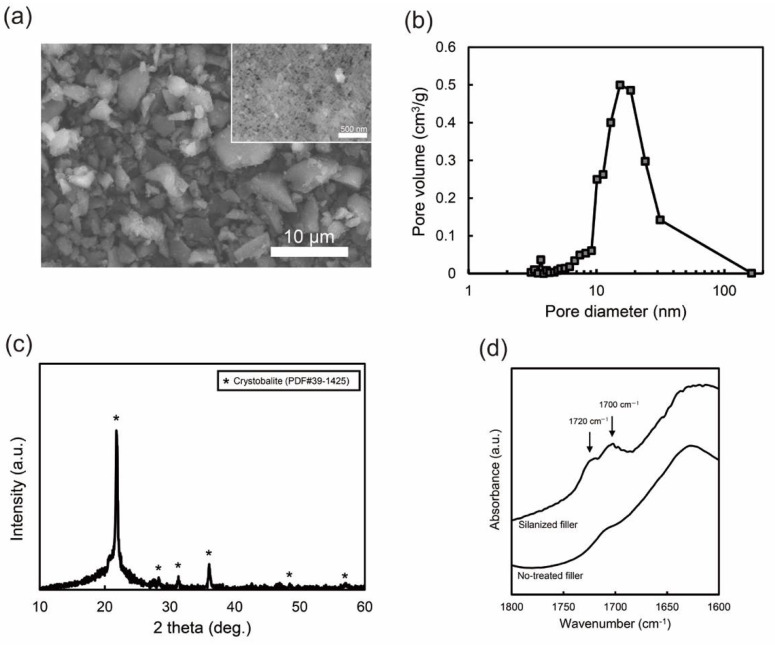
Characteristics of the porous silica filler: (**a**) SEM images for the filler with ×3000 magnification and for the filler surface with ×50,000 magnification (inset image), (**b**) pore size distribution obtained by the nitrogen desorption measurement, (**c**) XRD pattern, and (**d**) FT-IR spectra of the fillers with and without silanization.

The prepared filler was composed of irregular particles several micrometers in size and contained the nanopores (Figure 1a). The pore size and surface area were obtained from nitrogen sorption–desorption measurements. The BJH method gave the pore size distribution of the filler (Figure 1b). The pore size was in the range of approximately 5–30 nm and the mean diameter was 13.2 nm. The BET surface area of the filler was 61.3 m^2^/g. The crystal phase of the filler was determined using XRD (Figure 1c). The broad peak at ≈20° was attributed to the amorphous silica phase. In addition, sharp peaks were assigned to the cristobalite crystalline phase (PDF #39-1425). This XRD result suggests that the filler contained both amorphous and crystalline phases. The silanization of the filler was confirmed by FT-IR analysis in the range of 1600–1800 cm^−1^ (Figure 1d), where the characteristic peaks of the silane coupling agent are located [25,26,27]. The peak at approximately 1700 cm^−1^ was assigned to the stretching vibration of the carbonyl groups of the silane coupling agent [25,26,27]. Furthermore, the band at 1720 cm^−1^ was assigned to the hydrogen-bonded carbonyl groups of the silane coupling agent with the silanol groups on the silica [25,26,27]. The FT-IR results suggest that the filler surface was successfully coated with the silane coupling agent.

### 3.2. Filler-Loaded PMMA-Based Resin

**Figure 2:**
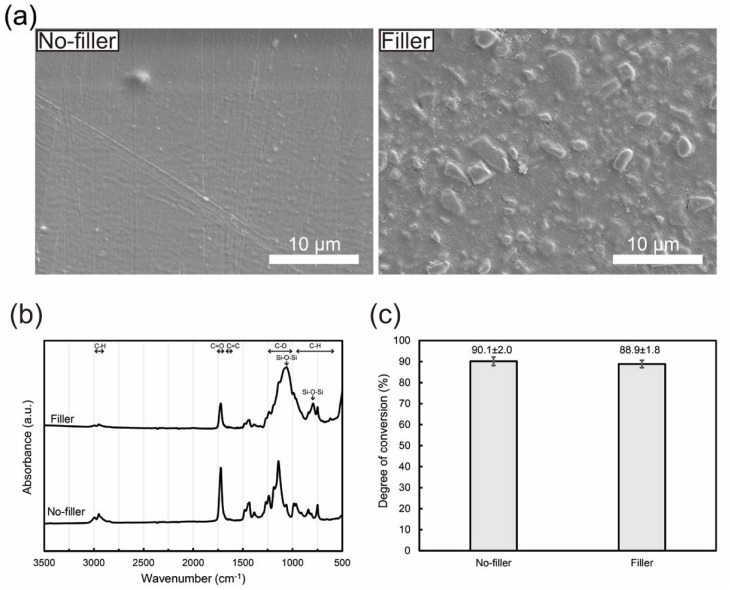
Characteristics of the PMMA-based resins with and without nanoporous silica filler: (**a**) SEM images, (**b**) FT-IR spectra, and (**c**) degree of conversions. The mean and standard deviation values are indicated in (**c**).

The PMMA-based resin after curing had a homogeneous morphology without any aggregates or voids (Figure 2a). The filler-loaded resin was a resin–ceramic composite with dispersed filler in a resin matrix (Figure 2a). Figure 2b shows FT-IR spectra of the PMMA-based resins with and without fillers. The FT-IR spectrum of the PMMA-based resin had characteristic peaks attributed to PMMA and poly-EGDMA, suggesting that the resin consisted of copoly(MMA/EGDMA) [28]. The peaks at 2900–3000 cm^−1^ were attributed to C–H stretching vibrations, 1250–1000 cm^−1^ to C–O stretching vibrations, and 950–650 cm^−1^ to C–H bending vibrations. The FT-IR spectrum of the filler-loaded PMMA-based resin also had characteristic peaks from silica, with a peak at ≈800 cm^−1^ for the Si–O–Si symmetric vibration, and a peak at ≈1020 cm^−1^ for the Si–O–Si asymmetric vibration [29,30]. Furthermore, the degree of conversion for each sample was estimated from the FT-IR spectra using the characteristic peaks of *C*=*C* bands at 1637 cm^−1^ and *C*=*O* bands at 1720 cm^−1^ for methacrylates. The calculated degree of conversion was 88.9 ± 1.8 and 90.1 ± 2.0% for PMMA-based resins with and without filler, respectively (Figure 2c). As there was no significant difference between these values, filler addition did not affect the degree of conversion of the PMMA-based resin.

**Figure 3:**
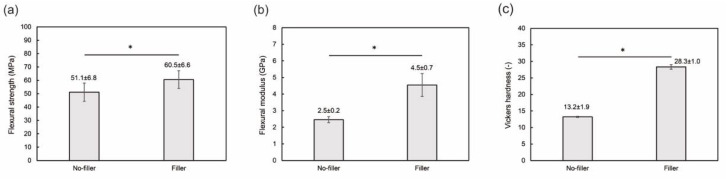
Mechanical properties of the PMMA-based resins with and without nanoporous silica filler: (**a**) flexural strength, (**b**) flexural modulus, and (**c**) Vickers hardness. The asterisk (*) indicates a significant difference between the values analyzed by Student’s *t*-test (n = 10, *p* < 0.01). The mean and standard deviation values are indicated in the figures.

**Figure 4:**
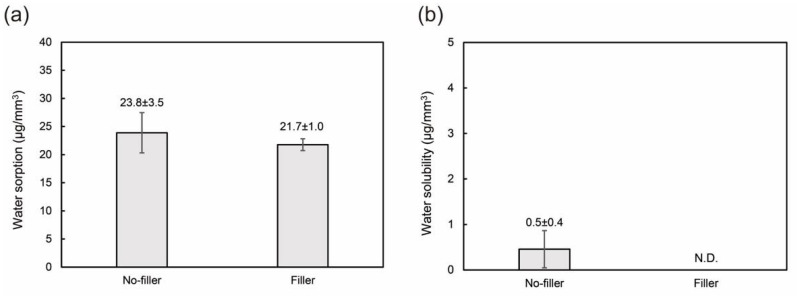
Physicochemical properties of the PMMA-based resins with and without nanoporous filler: (**a**) water sorption and (**b**) water solubility. The mean and standard deviation values are indicated in the figures.

The mechanical properties of PMMA-based resins are shown in Figure 3. The flexural strength, flexural modulus, and Vickers hardness values of the filler-loaded resin were significantly higher than those of the pure resin. Hence, the addition of the filler improved the mechanical properties of the PMMA-based resin. Figure 4 shows the physicochemical properties (water sorption and solubility) of the PMMA-based resins. There was no significant difference between the water sorption values. No water solubility was observed for the filler-loaded PMMA-based resin, whereas little solubility was confirmed for the pure resin. Therefore, filler addition improved the mechanical and physicochemical properties of the PMMA-based resins.

## 4. Discussion

The nanoporous silica filler was obtained via sintering, grinding, and silanization. During the sintering process, monolithic porous silica was produced by sintering the green body, containing the silica nanoparticles and PVA binder. During sintering, the PVA binder of the green body thermally decomposed, resulting in the formation of continuous pores in the body. Further details were reported in our previous study [31]. This previous study revealed that the porous structure can be controlled by the sintering temperature and time [31]; the pore volume and surface area decreased with increasing sintering temperature or time. Excessive sintering yielded a dense monolith with no pores, while insufficient sintering yielded a fragile monolith with poor consolidation of the nanoparticles. The sintering conditions were optimized to achieve a sufficiently porous and strong silica monolith. Then, the porous silica monolith was ground into a fine powder. Although the fabricated filler had microscale particles, it had a large surface area owing to the internal nanopores. This unique characteristic of the nanoporous filler significantly differs from that of conventional micro- or nano-fillers used in dental resin composites [32,33,34]. Furthermore, the present filler differs from the previously reported thermally sintered nanoporous silica filler in terms of its porous structure; the surface area of the present filler (61.3 m^2^/g) is eight times higher than that of an existing filler (8.4 m^2^/g) [19].

The resin matrix of the filler-loaded PMMA-based resin was prepared from appropriate amounts of pre-polymerized PMMA powder, MMA, EGDMA cross-linker, and BAPO photo-initiator. This system is similar to the powder–liquid mixtures used in many commercial self-cured acrylic resin systems [35,36]. In our system, the EGDMA cross-linker creates a three-dimensional polymer network with the MMA monomer via free-radical polymerization by light irradiation [37]. The pre-polymerized PMMA powder facilitated curing of the resin monomer. In our preliminary experiments, the resin without EGDMA and PMMA powder did not cure under light irradiation. Hence, EGDMA and PMMA powders are essential components for the present PMMA-based resin. The BAPO photo-initiator is commonly used for free-radical photopolymerization in biomedical resin-based applications owing to its low cytotoxicity and high absorption coefficient under ultraviolet light [38]. A small amount of initiator achieved an efficient degree of conversion in the PMMA-based resins. The degree of conversion reached ≈90% in the PMMA-based resins, regardless of filler addition. This suggests that the infiltrated resin inside the filler particles can be polymerized under light irradiation. The degree of conversion for the PMMA-based resins is close to that of commercial heat-polymerized PMMA-based resin (≈92–97%) [39], whereas it is higher than that of commercial photo-polymerized resin composites for dental bulk-filled applications (≈30–80%) [40]. Hence, PMMA-based resins are considered acceptable as photocurable resins. 

The PMMA-based resin contained 23 wt % filler. In this experiment, we examined the upper concentration limit at which the filler was able to disperse into the resin matrix without forming agglomerates or voids. We also prepared a filler-loaded PMMA-based resin using a non-silanized silica filler. In this case, the filler formed aggregates in the resin matrix at concentrations below 5 wt %. This means that silanization of the filler is essential and effective for homogeneous dispersion of the filler in the resin matrix. The present filler concentration (23 wt %) is higher than those of the previous filler-loaded PMMA-based resins listed in a review paper on filler-loaded PMMA-based resins [7]. The fillers often easily form aggregates in the resin matrix, and the upper limit of the filler load is approximately 5 wt % to avoid degradation of the mechanical properties, even when silanization of the filler is performed. In fact, we tried to use the original fumed silica nanoparticles as the filler for the PMMA-based resin. However, homogeneous filler-loaded resin was not successfully fabricated because the fumed silica formed aggregates in the resin matrix, even at a concentration below 5 wt %, owing to their high surface area of 300 m^2^/g. In contrast, the present micro-sized filler had a hierarchical structure containing nanopores. This unique microstructure of the filler allows the resin matrix to penetrate the continuous nanopores via capillary forces, leading to good wettability between the filler and resin matrix. This inhibits the aggregation of the fillers and void formation in the resin matrix. 

The mechanical properties of the pure PMMA-based resin are comparable to those of commercial self-cured PMMA-based resins [41]. The addition of the filler significantly increased the mechanical properties of the PMMA-based resin. In particular, the flexural modulus and Vickers hardness of the filler-loaded PMMA-based resin were twice those of the pure PMMA-based resin. These improvements in the mechanical properties are notable in comparison with previous studies on filler-loaded PMMA-based resins. For instance, nanosized ZrO_2_ filler added at 7 wt % increased the Vickers hardness only marginally, from 17.3 to 19.6 [42]. In another study, the addition of aluminum borate whiskers at 20 wt %, increased the Vickers hardness of the resin from 17.78 to 20.58 [43]. 

The physicochemical properties of the PMMA-based resin also improved with the addition of the filler. This suggests that the filler particles were well dispersed in the resin matrix and chemically bonded to each other. A previous study suggested that the water solubility and sorption of resin composites are affected by the material homogeneity [44], where highly homogeneous resin–filler composites have lower water solubility and sorption. In addition, the silane coupling agent on the filler surface can improve the bonding between the filler and resin matrix, thereby enhancing the mechanical and physicochemical properties [45,46]. The mechanical and physicochemical properties of filler-loaded PMMA-based resin meet the criteria of ISO standard for polymer-based crown [22]. Overall, the filler-loaded PMMA-based resin is considered suitable for use in dental applications, such as temporary crowns, provisional restorations, and denture bases. However, there is scope for further assessment of the cytotoxicity of PMMA-based reins to confirm their biocompatibility using human gingival fibroblasts and gingival keratinocytes [47] and in vivo allergy tests [48]. Since TEGDMA has a risk of disruption of vital cell functions, the cytotoxicity due to the unpolymerized monomers will be examined further [49,50].

The present study demonstrated that the developed nanoporous silica filler can improve the mechanical and physicochemical properties of PMMA-based resins. In addition, the nanoporous silica filler has potential applications in other resins, such as multifunctional resins, urethane dimethacrylate, and bisphenol A-glycidyl methacrylate. Multifunctional resins have been widely used as resin composites for dental restorative materials owing to their excellent mechanical properties and biocompatibility. Commercial resin composites are generally loaded with nano- or micro-sized fillers to improve their mechanical properties [51]. The nanoporous silica fillers further improved the mechanical properties of the resin composites. The filler size and porous structure would be controlled to enable good dispersion in the resin matrix to achieve desirable properties. The potential applications of the newly developed filler will be examined in the future to expand the use of nanoporous fillers in clinical applications.

## 5. Conclusions

A nanoporous silica filler was prepared via a sintering process. The filler was incorporated in the photocurable PMMA-based resin to improve the mechanical and physicochemical properties. The filler-loaded PMMA-based resin has the potential for use in dental restorative materials.

## Figures and Tables

**Table 1:** Reagents used for sample preparation.

Acronym	Reagent Name	Purpose	Purity (%)	Supplier
SiO_2_	Fumed silica (Aerosil^®^ 300)	Ceramic	99.8	Nippon Aerosil Co., Ltd., Tokyo, Japan
PVA	Poly(vinyl alcohol)	Binder	78–82 *	Fujifilm Wako Pure Chemical Corporation, Osaka, Japan
γ-MPTS	3-Methacryl oxypropyl trimethoxysilane	Silane coupling agent	99.9	Shin-Etsu Chemical Co., Ltd., Tokyo, Japan
PMMA	Polymethyl methacrylate	Polymer	Mw ≈ 15,000	Sigma-Aldrich Co. LLC., St. Louis, MS, USA
MMA	Methyl methacrylate	Monomer	>98	Fujifilm Wako Pure Chemical Corporation, Osaka, Japan
EGDMA	Ethylene glycol dimethacrylate	Cross-linker	>97	Tokyo Chemical Industry Co., Ltd., Tokyo, Japan
BAPO	Phenylbis (2,4,6-trimethylbenzoyl) phosphine oxide	Photo-initiator	>96	Tokyo Chemical Industry Co., Ltd., Tokyo, Japan

* Degree of saponification.

## Data Availability

The data presented in this study are available on request from the corresponding author.
